# Equity in health care: An urban and rural, and gender perspective; the Suriname Health Study

**DOI:** 10.3934/publichealth.2018.1.1

**Published:** 2018-02-27

**Authors:** CCF Smits, JR Toelsie, MGM Eersel, ISK Krishnadath

**Affiliations:** 1 Faculty of Medical Sciences, Department of Public Health, Anton de Kom University of Suriname; 2 Faculty of Medical Sciences, Department of Physiology, Anton de Kom University of Suriname

**Keywords:** equity, healthcare use, gender differences, urban, rural coastal and rural interior, socio-economic factors, perceived illness

## Abstract

**Background:**

The literature reports that the use of healthcare services in urban areas compared to rural areas and by females compared to males is often higher. The aim of this study is to evaluate equity on geographical living area and gender for the use of primary and secondary healthcare in Suriname.

**Methods:**

We used 5,671 records (99%) from the Suriname Health study which was designed according to World Health Organization (WHO) Steps guidelines. We evaluated the Prevalence Ratio (PR) for living area and gender in using primary (PHC) and secondary healthcare (SHC) adjusted for the perceived need for healthcare, socio-economic factors and disease factors and the effect of all factors was measured.

**Results:**

Overall a percentage of 46.7 (95% Confidence Interval (CI) 45.1–48.4) had used primary healthcare and 12.7 (95% CI 11.6–13.8) secondary healthcare in the past 12 months. The PR for males compared to females was 0.75 (95% CI 0.70–0.81) for primary healthcare and 0.82 (95% CI 0.69–0.98) for secondary healthcare. The PR for urban and rural coastal areas compared to the rural interior was 1.52 (95 % CI 1.36–1.70) and 1.53 (95% CI 1.36–1.71), respectively. For the use of SHC, the PR for urban and rural coastal areas compared to the rural interior was 9.3 (95 % CI 5.44–15.89) and 8.58 (95% CI 4.98–14.81). The attributable effect of perceived healthcare-need to the PR of the urban and rural coastal areas was 39.64% and 37.81% compared to the rural interior for secondary healthcare. Further, 31.74% and 13.56% were due to socioeconomic factors.

**Conclusion:**

Although we observed equity between living areas for PHC use, for SHC use we observed a disadvantaged position for the rural interior, mainly influenced by socioeconomic factors. We measured gender equity for both PHC and SHC use.

## 1. Introduction

Equity in health, defined as the absence of avoidable or remediable differences among groups of people [1], is considered a major public health issue in developed [2],[3] as well as in developing countries [4],[5]. It challenges healthcare systems and policy makers to ensure optimal access to healthcare and to address causes of disadvantages. In general, disadvantaged groups are recognized by social, racial, ethnic, economic or demographic differences [6] that are known to negatively impact their health [7].

Since 1948, the WHO has recognized health as a fundamental right of every human being without distinction of race, religion, and political belief, economic or social condition [8]. Thereafter, the WHO has endorsed several programs to improve equity in health [9]. In 1996 the Global Health Equity Initiative and in 2005, the Commission on Social Determinants were established to promote equity in health [9]. Several issues such as gender, social determinants, ethics, health care financing, globalization, and policy were addressed [10]. It was revealed that gender inequities are present in all societies and effect health outcomes [11]. Gender related inequity in health can be related to biological or social factors. The social determinants such as the circumstances in which people are born, grow, live, work and age contribute to a high burden of illness and premature death [11]. In developing countries, enormous differences exist between the poorer rural and the richer urban areas. It is known that 99% of all maternal death occurs in developing countries, which are usually challenged by their limited resources [12].

The Republic of Suriname, an upper-middle income country, located on the northeast of South America, has a multi-ethnic and multicultural population consisting mainly from Indian, African, and Indonesian descent. Approximately 90% of the population lives in the urban and rural coastal areas where public and private primary and secondary health care services are extensively available. For residents of the rural interior, which is covered in tropical rainforest, free primary health care is provided through 56 clinics, nearby every main village and run by a government subsidized NGO (Medical Mission) [12]. Selected secondary services by means of outreach missions and referral to further specialist care in the coast are also available [12]. The Gross Domestic Product (GDP) was 3.62 billion US dollars in 2016 [13]. The unemployment rate is 8.4% in the urban areas and increases up to 16.7% in the rural areas. Less than half of the women between 15 and 65 years are economically active and only 13.7% of them are employed [14].

The government of Suriname moves towards implementation of the “Health in All Policies” in order to improve equity in health [14]. However, as far as we know, no data on equity with respect to the use of healthcare are available. Therefore, we evaluated equity on geographical living area and gender for the use of primary and secondary healthcare. We used data from the Suriname Health Study, a nationwide study on non-communicable disease (NCD) risk factors, to assess equity in health.

## 2. Methods

As described previously, we used data of the Suriname Health Study [15], a cross sectional population study, designed according to WHO Steps guidelines [16] and approved by the Ethics Committee of the Ministry of Health [15]. This study applied a stratified multistage cluster sample of households to select respondents between March and September of 2013. In total, 343 clusters were selected randomly within the enumeration areas of the ten districts of Suriname. In the selected househould, the respondent was identified with a Kish grid [17], a pre-assigned table with random numbers. Respondents were informed about the details of the study and were requested to sign for consent.

### 2.1. Outcome factors

We analyzed the use of primary and secondary healthcare services reported in the last 12 months. The use of healthcare was measured by at least one reported visit to a health facility. While healthcare use reflects the actual access to health care and the availability of services, the need for health care was self-reported. Apart from residential area, we included sex, possession of health insurance, educational level and income status in the analysis. Further, the self-reported presence of diabetes, hypertension or other chronic diseases was included. The residential addresses were stratified into urban, rural coastal areas and the rural interior. Educational levels were divided into low (primary school education or lower), middle (middle or secondary education) and high (above middle or secondary) education. The wealth index was classified as the income status quintile from the Ministry of Internal Affairs of Suriname in Surinamese dollars, SRD (1USD = 3.35SRD). The 1st quintile corresponded to the lowest income and the 5th to the highest.

### 2.2. Statistical analysis

All collected data were subjected to a weighting procedure so inferences could be made to the whole population. The weights used for analysis were calculated to adjust for probability of selection, non-response and differences between the sample population and target population. We used the weighted data first, to calculate the proportions of the population overall, per residential area and by sex (Table 1). Second, we used poisson regression models to examine the prevalence ratios (PR's) comparing the use of primary and secondary healthcare of those living in urban, rural coastal and rural interior and of men and women. We calculated the crude PR and adjusted for the self-reported need to use healthcare, socio-economic factors (Wealth index, Education and Health Insurance), and for the self-reported presence of chronic disease including diabetes and hypertension (Table 2). Third, we calculated the attributable fraction of the perceived need of healthcare in the past 12 months. For risk factors (PR > 1) the formula (adjusted PR-1)/(Crude (or basic) PR-1) and for protective factors (PR < 1) the formula (1-adjusted PR)/(1-Crude (or basic) PR) was used. Finally, in Table 3, we calculated the attributable fraction of gender, socioeconomic and chronic disease related factors on the basic PR of secondary health care use adjusted for the reported need for healthcare. We used the Stata 12 for the statistical analyses.

## 3. Results

Table 1 reports the proportion of the PR for the use of healthcare and various related factors per living area and gender. Overall, 46.7% (95% CI 45.1–48.4) visited primary healthcare and 12.7% (95% CI 11.6–13.8) secondary healthcare facilities in the past 12 months. For both primary and secondary healthcare most visits occurred in the urban and rural coastal areas compared to the rural interior and in women compared to men.

The perceived need for healthcare in the last 12 months was more frequent in the urban 53.6% (95% CI 51.5–55.7) and rural coastal areas 51.9% (95% CI 49.2–54.6) compared to the rural interior 34.7% (95% CI 31.4–38.0) and in women 58.6% (95% CI 56.6–60.6) compared to men 45.0 % (95% CI 42.4–47.5). Also, the presence of self-reported chronic diseases, including diabetes and hypertension, was more frequent in the urban and rural coastal areas compared to the rural interior and in women compared to men. The coverage for health insurance was higher in the rural interior areas compared to urban and rural coastal areas and was higher in women compared to men. For higher education the highest percentage 23.2% (95% CI 21.5–25) was found in the urban areas and for lower education the highest percentage 92.6% (95% CI 90.8–94.3) was in the rural interior. There is no statically significant difference between the percentages for lower education in men 53.3% (95% CI 50.7–55.9) and women 52.5 % (95% CI 50.4–54.6). For higher education the highest percentage 20.8% (95% CI 19.1–22.6) was found in women.

For the wealth index the highest percentage for the lowest quintile was found in the rural interior 72.5% (95% CI 68.6–76.4) and in women 39.0% (95% CI 36.5–41.5), whilst the highest quintile was observed in urban areas 13.0% (95% CI 11.1–14.8) and men 12.7 % (95% CI 10.3–15).

Table 2 shows the use of primary and secondary healthcare use in urban and rural coastal living areas compared to rural interior areas and for women compared to men. The crude PR in the use of primary healthcare is statistically significant (*p* < 0.05) for urban 1.52 (95% CI 1.36–1.70) and rural coastal living areas 1.53 (95% CI 1.36–1.71) compared to rural interior areas and for men 0.75 (95% CI 0.70–0.81) compared to women. For the use of secondary healthcare, the PR increased to 9.3 (95% CI 5.44–15.89) for urban living areas and for rural coastal living areas 8.59 (95% CI 4.98–14.82) compared to rural interior areas and decreased between women and men 0.82 (95% CI 0.69–0.98). The PR for men compared to women did not change when adjusted for living area, nor did the PR for the rural living areas compared with the urban area change when adjusted for gender. However, when adjusted for the perceived need of healthcare use, socio-economic factors and the self-reported presence of chronic disease there was no difference in use for healthcare in all living areas nor between men and women. For secondary healthcare use the differences between living areas changed marginally when adjusted for gender but reduced considerably when adjusted for the other factors.

The attributable fraction of the perceived need for healthcare in the crude PR comparing urban with rural interior was 39.64% and 37.81% when comparing rural coastal with rural interior.

In Table 3 we observed attributable risk on the PR for the use of secondary healthcare adjusted for the perceived need for healthcare. The attribution of gender was 0.4% in the comparison of urban with rural interior and 0.64% when comparing rural coastal with rural interior. The attribution of socio-economic factors was 31.74% in the comparison of urban with rural interior and 13.56% when comparing rural coastal with rural interior. The knowledge of having a chronic disease did not attribute to the comparison of urban with rural interior but did so for 0.64% when comparing rural coastal with rural interior.

**Table 1.** The distribution of healthcare usage, disease perceptions, and socio-economic factors by living area and gender.

	Total N = 5,671 % (CI 95%)	Urban N = 2,767 % (CI 95%)	Rural coastal N = 1,947 % (CI 95%)	Rural interior N = 957 % (CI 95%)	Female N = 3,555 % (CI 95%)	Male N = 2,116 % (CI 95%)
Use of primary HC	46.7 (45.1–48.4)	48.0 (46.0–50.1) ^a^	48.3 (45.1–50.9) ^a^	31.6 (28.4–34.8) ^b^	53.3 (51.3–55.4)ˣ	40.1 (37.6–42.6)ˠ
Use of secondary HC	12.7 (11.6–13.8)	13.9 (12.5–15.2) ^a^	12.8 (11.0–14.6) ^a^	1.5 (0.7–2.3)^b^	13.9 (12.5–15.3)ˣ	11.4 (9.8–13.1)ˠ
Perceived need for HC	51.8 (50.2–53.4)	53.6 (51.5–55.7) ^a^	51.9 (49.2–54.6) ^a^	34.7 (31.4–38.0) ^b^	58.6 (56.6–60.6)ˣ	45.0 (42.4–47.5)ˠ
Self-reported chronic disease	19.8 (18.6–21.1)	20.2 (18.7–21.8) ^a^	20.2 (18.1–22.3) ^a^	14.9 (12.5–17.3) ^b^	21.5 (19.9–23.1)ˣ	18.1 (16.2–20)ˠ
Self-reported diabetes	7.9 (7.1–8.8)	8.4 (7.3–9.5) ^a^	8.9 (7.5–10.4) ^a^	1.8 (0.9–2.6) ^b^	8.9 (7.8–9.9)ˣ	7.0 (5.7–8.3)ˠ
Self-reported hypertension	18.3 (17.1–19.5)	18.6 (17.1–20.1) ^a^	19.2 (17.1–21.2) ^a,b^	13.6 (11.3–15.9) ^c^	21 (19.4–22.6)ˣ	15.6 (13.8–17.4)ˠ
Health insurance	80.0 (78.7–81.3)	79.0 (77.2–80.7) ^a^	81.5 (79.4–83.6) ^a^	86.1 (83.6–88.6) ^b^	87.2 (85.7–88.6)ˣ	72.7 (70.4–75.1)ˠ
Education level						
* Low*	52.9 (51.2–54.6)	46.2 (44.1–48.3) ^a^	63.6 (60.9–66.2) ^b^	92.6 (90.8–94.3) ^c^	52.5 (50.4–54.6)	53.3 (50.7–55.9)
* Middle*	27.9 (26.4–29.5)	30.6 (28.7–32.5) ^a^	26.8 (24.3–29.3) ^a^	6.3 (4.6–7.9) ^b^	26.6 (24.7–28.5)ˣ	52.5 (50.4–54.6)ˠ
* High*	19.2 (17.8–20.5)	23.2 (21.5–25) ^a^	9.6 (8.1–11.1) ^b^	1.1 (0.5–1.8) ^c^	20.8 (19.1–22.6)ˣ	17.5 (15.4–19.5)ˠ
Wealth Index						
* 1*	33.6 (31.7–35.5)	28.5 (26.1–30.9) ^a^	32.7 (29.5–36) ^a^	72.5 (68.6–76.4) ^b^	39.0 (36.5–41.5)ˣ	28 (25.1–30.9)ˠ
* 2*	33.7 (31.7–35.7)	34.6 (32.1–37.2) ^a^	37.7 (34.4–41) ^a^	20.5 (17–24) ^b^	32.5 (30.1–35)	34.9 (31.7–38.2)
* 3*	15.1 (13.6–16.7)	16.2 (14.2–18.2) ^a^	17.2 (14.5–20) ^a^	3.6 (1.9–5.3) ^b^	14.2 (12.3–16.1)	16.1 (13.6–18.5)
* 4*	6.8 (5.7–7.9)	7.7 (6.2–9.1) ^a^	6.2 (4.5–7.9) ^a^	1.3 (0.3–2.4) ^b^	5.3 (4.1–6.5)	8.3 (6.4–10.2)
* 5*	10.8 (9.4–12.2)	13.0 (11.1–14.8) ^a^	6.1 (4.5–7.7) ^b^	2.1 (0.8–3.4) ^c^	9.0 (7.4–10.6)ˣ	12.7 (10.3–15)ˠ

HC: Healthcare; The matching subscript letters a, b, and c denote a subset of living areas categories whose column proportions do not differ significantly from each other at the 0.05 level; For gender the symbols ˠ and ˣ denote a subset of categories whose column proportions differ significantly from each other at the 0.05 level.

**Table 2.** The Prevalence Ratio for the use of primary and secondary healthcare by living area and gender adjusted for each other and for the perceived need for healthcare, socio-economic factors and the presence of chronic diseases.

Living area	Healthcare use	Gender	Need for healthcare	Socio-economic factors	Chronic disease related	All factors
	PR (IC 95%)	PR (IC 95%)	PR (IC 95%)	PR (IC 95%)	PR (IC 95%)	PR (IC 95%)
Primary healthcare use	*p* = 0.0000	*p* = 0.0000	*p* = 0.0378	*p* = 0.0102	*p* = 0.0436	*p* = 0.0187
*Rural interior areas*	1.00	1.00	1.00	1.00	1.00	1.00
*Urban areas*	1.52 (1.36–1.7)	1.53 (1.38–1.71)	0.98 (0.94–1.03)	1.03 (0.96–1.1)	0.98 (0.94–1.03)	1.02 (0.95–1.1)
*Rural coastal areas*	1.53 (1.36–1.71)	1.55 (1.36–1.73)	1.02 (0.98–1.06)	1.07 (1.01–1.14)	1.02 (0.97–1.06)	1.07 (1–1.14)
Secondary healthcare use	*p* = 0.0000	*p* = 0.0000	*p* = 0.0000	*p* = 0.0002	*p* = 0.0000	*p* = 0.0001
*Rural Interior areas*	1.00	1.00	1.00	1.00	1.00	1.00
*Urban areas*	9.3 (5.44–15.89)	9.4 (5.48–16.0)	6.01 (3.55–10.19)	4.42 (2.03–9.64)	6.01 (3.56–10.17)	4.43 (2.04–9.62)
*Rural coastal areas*	8.59 (4.98–14.82)	8.67 (5.03–14.96)	5.72 (3.35–9.76)	5.08 (2.34–11.04)	5.69 (3.33–9.7)	5.15 (2.37–11.15)

Gender	Healthcare	Living area	Need for healthcare	Socio-economic factors	Chronic disease related	All factors
	PR (IC 95%)	PR (IC 95%)	PR (IC 95%)	PR (IC 95%)	PR (IC 95%)	PR (IC 95%)
Primary healthcare use	*p* = 0.0000	*p* = 0.0000	*p* = 0.1941	*p* = 0.8449	*p* = 0.2532	*p* = 0.9306
*Female*	1.00	1.00	1.00	1.00	1.00	1.00
*Male*	0.75 (0.7–0.81)	0.75 (0.69–0.81)	0.98 (0.95–1.01)	1 (0.95–1.04)	0.98 (0.95–1.01)	1 (0.96–1.04)
Secondary healthcare use	*p* = 0.0288	*p* = 0.0000	*p* = 0.4030	*p* = 0.6251	*p* = 0.4800	*p* = 0.6660
*Female*	1.00	1.00	1.00	1.00	1.00	1.00
*Male*	0.82 (0.69–0.98)	0.81 (0.68–0.97)	1.07 (0.91–1.26)	1.06 (0.84–1.33)	1.06 (0.9–1.25)	1.05 (0.84–1.31)

Socio-economic factors include health insurance; education and wealth index.

Chronic disease related include self-reported diabetes, hypertension and other chronic diseases.

**Table 3.** The attributable risk fraction for the crude use of secondary healthcare adjusted for the perceived need of healthcare.

Secondary HC^a^	PR Basic	Gender		Socio-economic factors		Chronic disease factors	
		PR	% attributed	PR	% attributed	PR	% attributed
Rural interior	1.00	1.00		1.00		1.00	
Urban	6.01	5.99	0.40	4.42	31.74	6.01	0.00
Rural coastal	5.72	5.69	0.64	5.08	13.56	5.69	0.64

Secondary HC^a^: Secondary healthcare adjusted for perceived need. Socio-economic factors include health insurance; education and wealth index. Chronic disease related factors include self-reported diabetes, hypertension and other chronic diseases.

## 4. Discussion

In this study, we evaluated equity on geographical living area and gender for the use of primary and secondary health care in Suriname. We found that the use of primary health care in urban and rural coastal areas were alike. In contrast, the use of secondary health care was lower in the rural interior and about 40% of this difference was attributed to the perceived need. From there, socio-economic factors explained the difference between urban and rural interior areas and between rural coastal and rural interior areas for about 30% and 14%, respectively. We observed that women use health care facilities more frequently than men do. However, these differences were no longer significant when adjusted for the perceived need of health care, socio-economic factors and the self-reported presence of chronic disease.

In Latin America challenges remain concerning inadequate decentralization of health services, particularly in remote areas, persisting socio-economic disparities and inefficiencies in the health care systems which hamper an equitable distribution of health care services across socio-economic strata and geographical regions [18],[19]. Progress has been made in this region since the 1980s in scaling up health care coverage and specifically improving access to integrated primary health care services [19]. Our findings showed that nationally the health insurance coverage was around 80% but in the rural interior it was statistically significantly higher compared to the rest of the country. This is due to government policy, which dates from the 1960s that people residing in the rural interior have free access to local primary health care and referral to secondary (specialist) care in the coast. This arrangement is quite unique, particularly in the Latin American region. Despite the high coverage of health insurance in the rural interior, the perceived need for health care and the actual use of health care services were statistically significantly lower in the rural interior compared to the urban and rural coastal areas. These differences were more profound when comparing the use of secondary (specialist) health care services. The large difference in the use of secondary (specialist) care services could be partly explained by the fact that health care workers in the remote polyclinics of the Medical Mission consult specialists by telephone or short wave radio for advice and instructions [12]. Patients are only transported to the coastal area for face-to-face specialist consultation, when necessary [12]. Travelling to the coastal area and back home can be very costly for the Medical Mission and for the populations in the rural interior districts who carry a disproportionate burden of poverty compared to those living in the urban and rural coastal areas [20].

The lower perceived need and actual use of health care services in the rural interior, despite the higher health care coverage could partly be explained by a lower chronic disease burden illustrated by the relatively statistically significantly lower prevalence of self-reported diabetes and hypertension. Studies have shown declining rates of infectious diseases, especially malaria, over the past decades in the rural interior, and consequently a dwindling annual average number of polyclinic visits, which can be attributed to successful antimalaria and other infectious disease programs [21],[22]. On the other hand, there still is a relatively low, though slowly increasing, burden of chronic diseases (diabetes, hypertension) which could be attributed to the more traditional lifestyle of rural interior populations consisting of hunting, fishing and gardening [23]–[25]. Also the perceived need of disease is subjective and related to the availability of services [26]. However, the further development of healthcare services to meet the emerging health needs, especially related to ageing and chronic non-communicable diseases, is not only a priority for the rural interior but also for the rest of Suriname as for all regions that underwent a rapid epidemiological and demographic transition [19].

Gender differences in health status as well as in utilization of health care services have been described previously in the literature [27]–[35]. A wide range of psychological, behavioral and socio-economic factors contribute to these disparities. Studies have indicated that women experience poorer health [27],[28],[30],[32], which seems to be associated with their higher morbidity burden [27] which then translates into higher health care needs and utilization of health care services [36],[37]. The WHO links women's poorer health status on poverty, intimate partner violation and gender power relations who are not in favor of women [10],[11],[37]–[41]. Studies also show that single female-headed families are more vulnerable to poverty and consequently to health risks [42]–[50]. In Suriname gender differences have been described in a study of patient records from primary health care clinics in the coastal area of Suriname. Women were twice more likely than men to visit the clinics for diabetes and three times as likely for hypertension or a combination of diabetes and hypertension [51]. All of the above mentioned factors may have affected the higher perceived need for health care among women in our study, which on its turn could explain their higher use of primary health care facilities in particular [36],[52].

As for men, their willingness to seek help seems to be negatively affected by many factors. Most of these are psychological and can be ascribed to adherence to traditional masculinity norms, for example embarrassment, symptom minimization, regarding one as not being susceptible to disease, and restricted emotional expression [29],[53]–[55] In Suriname men tend to visit health care facilities less frequently than women [51]. This is, for instance, reflected in the fact that differences between self-reported and measured hypertension and diabetes mellitus are more common among men than women [56],[57]. The gender differences found in our study are in line with these findings. Compared to women, men in our study visited significantly less frequent a general practitioner. These differences are explained by socio-economic factors, perceived health care need and self-reported chronic diseases. Additional factors that could help to explain the differences, like ethnicity, age, employment status, male versus female socialization, education and psychological factors were not included in our study [10],[11],[37],[58]. Exploring differences in perceived need between men and women in Suriname therefore remains subject to further research.

The strength of this cross-sectional study was the design with a stratified multistage cluster, adequate to represent the ethnic and geographic diversity within the Surinamese population by sex in 5 different age-groups [15]. The use of trained interviewers, the inclusion of control questions in the questionnaire and the intense monitoring on consistency and completeness that included random checks on responses of participants improved the validity of our self-reported data [15]. In addition, sample weights were applied in the analysis to correct for selection and response bias. In general, the percentage of missing data, was relatively small (<2%), except for the information on income status.

Still, some limitations should be considered. First, the different healthcare systems available and coping strategies with diseases, of the different people in the various living areas were not evaluated. Second, although the wide range of confounding variables are evaluated in this study many are also missing for example family ties and available social support systems. Third the reporting on use of health care can be subject to recall bias. Finally, all variables were self-reported making the information on the perceived need for healthcare and the presence of disease highly subjective and less reliable.

## 5. Conclusions

The results of this study show equity between the different living areas for the use of primary health care. However, the populations of the rural interior experienced a disadvantaged position with regard to the use of secondary health care, largely influenced by socio-economic factors.

Differences between women and men can be attributed to perceived need of healthcare use, socio-economic factors and self-reported presence of chronic diseases.

To better understand the complex and oftentimes entwined linkages between social, economic, psychological, cultural and biological circumstances of people, more research is needed to further explain the differences found in health care use between living areas and gender in Suriname.

Also research on perceived need and health care use in various health care systems is required to improve the quality and responsiveness of the health care services.

It is evident that health is not just the responsibility of the health sector, but the result of all public policies. The implementation of the ‘Health in All Policies’ approach is a commendable initiative of the Surinamese government. Based on the results of this study we advise to focus expenditures and programs on the most deprived populations and areas to alleviate structural poverty and improve health equity.
